# Equine Stereotaxtic Population Average Brain Atlas With Neuroanatomic Correlation

**DOI:** 10.3389/fnana.2019.00089

**Published:** 2019-10-03

**Authors:** Philippa J. Johnson, Valentin Janvier, Wen-Ming Luh, Marnie FitzMaurice, Teresa Southard, Erica F. Barry

**Affiliations:** ^1^Department of Clinical Sciences, Cornell College of Veterinary Medicine, Cornell University, Ithaca, NY, United States; ^2^Cornell Magnetic Resonance Imaging Facility, Cornell College of Human Ecology, Cornell University, Ithaca, NY, United States; ^3^Department of Biomedical Sciences, Cornell College of Veterinary Medicine, Cornell University, Ithaca, NY, United States

**Keywords:** ANTs, volume, gray matter, white matter, laterality, segmentation, tissue segmentation maps

## Abstract

There is growing interest in the horse for behavioral, neuroanatomic and neuroscientific research due to its large and complex brain, cognitive abilities and long lifespan making it neurologically interesting and a potential large animal model for several neuropsychological diseases. Magnetic resonance imaging (MRI) is a powerful neuroscientific research tool that can be performed *in vivo*, with adapted equine facilities, or *ex-vivo* in the research setting. The brain atlas is a fundamental resource for neuroimaging research, and have been created for a multitude animal models, however, none currently exist for the equine brain. In this study, we document the creation of a high-resolution stereotaxic population average brain atlas of the equine. The atlas was generated from nine unfixed equine cadaver brains imaged within 4 h of euthanasia in a 3-tesla MRI. The atlas was generated using linear and non-linear registration methods and quality assessed using signal and contrast to noise calculations. Tissue segmentation maps (TSMs) for white matter (WM), gray matter (GM) and cerebrospinal fluid (CSF), were generated and manually segmented anatomic priors created for multiple subcortical brain structures. The resulting atlas was validated and correlated to gross anatomical specimens and is made freely available at as an online resource for researchers (https://doi.org/10.7298/cyrs-7b51.2). The mean volume metrics for the whole brain, GM and WM for the included subjects were documented and the effect of age and laterality assessed. Alterations in brain volume in relation to age were identified, though these variables were not found to be significantly correlated. All subjects had higher whole brain, GM and WM volumes on the right side, consistent with the well documented right forebrain dominance of horses. This atlas provides an important tool for automated processing in equine and translational neuroimaging research.

## Introduction

The horse *(Equus caballus)* is one of the largest domesticated species and has a well-studied and historically close affinity with humans (Cozzi et al., 2014). There is growing interest in the horse for behavioral, neuroanatomic and neuroscientific research due to its large and complex brain, cognitive abilities and long life span making it neurologically interesting and a potential large animal model for several neuropsychological diseases (Cozzi et al., 2014; Roberts et al., 2017). In addition, as the use of high field magnetic resonance imaging (MRI) increases in veterinary hospitals (Manso-Díaz et al., 2015), development of tools that could be utilized to evaluate the equine brain and naturally occurring neuropathology is needed to advance equine neurology in the clinical environment.

Rodents are the most common animal models used in neuroscientific research, however, their brain structure and volume are significantly different to the human brain limiting their utility in this role. Therefore in the current translational neuroscience literature there is a need to identify novel animal models with more similar morphological brain features to humans with similar naturally occurring pathologies. In contrast to rodents, humans have a large brain with a high degree of cortical folding (gyrification index of 2.56; Zilles et al., 1988). The adult horse brain weighs on average 606.07 g and has a highly convoluted and complex pattern of sulci and gyri (Cozzi et al., 2014). The gyrification index for the horse is one of the highest in domesticated animals (documented as 1.99–2.80; Zilles et al., 2013). The large and complex nature of the horse brain provides distinct neuroanatomical similarities to humans, that rodent models may lack (rat gyrification index 1.02; Zilles et al., 2013). In addition, the horse exhibits testable cognitive function being able to the perform learning, discrimination, match-to-sample and memory tasks commonly used in neurological testing and automated systems have been developed to test equine cognitive function (Roberts et al., 2017).

In slowly-progressive diseases that occur in the aged brain, such as Huntington’s disease, ideal models need to have a long life-span in order to allow for extended study of the condition over a similar timeframe to that which the human condition would develop (Morton and Howland, 2013). Horses are the longest lived of the common domestic species, with life spans of 25–30 years. This extended life span makes them suited for the study of slowly progressive, later stage, neurological diseases, such as Huntington’s and Parkinson’s disease (Morton and Howland, 2013; Roberts et al., 2017). Horses also exhibit naturally occurring neurophysiological diseases that are similar to diseases in humans. One such condition is the equine psychological disorder of oral stereotypy (crib-biting) which has been proposed as a potential model for Tourette’s Syndrome in humans (Hemmings et al., 2007; Roberts et al., 2017).

In the veterinary clinical setting, evaluation of the brain using MRI is starting to become routine for work up of neurological disease (Manso-Díaz et al., 2015; Pease et al., 2017). Unfortunately, despite horses presenting with severe, and often career ending, neurological signs, such as seizures, narcolepsy and vestibular signs, 70% of MRI examinations have no visible abnormalities on standard MRI sequences and therefore there is a need to apply more advanced MRI methods to improve diagnosis and understanding of the pathophysiology of equine neurological disease (Manso-Díaz et al., 2015).

With growing interest in the equine brain as a model for human disease and a clinical need to apply advanced MRI techniques for neurological work ups, it is vital that tools are developed to assist in assessing their brain structure and function. MRI is a powerful neuroscientific research tool, that can be performed *in vivo* in the clinical setting with adapted equine facilities (Manso-Díaz et al., 2015), and *ex-vivo* in the research setting. Advanced MRI techniques, such as voxel-based morphometry and diffusion tensor imaging, provide novel methods for evaluation of the brain, improving our ability to detect neuropathology and understand how different pathologies affect the brain. They are routinely applied in the evaluation of disease processes in the research setting and have the potential to improve diagnosis and assessment of equine neurological disease clinically.

The brain atlas is a fundamental resource for advanced neuroimaging work, being vital for assessment of *in vivo* and *ex-vivo* imaging data (Ullmann et al., 2015). Brain atlases have been created for a multitude species involved in neurological research including the cat (Stolzberg et al., 2017), dog (Datta et al., 2012), sheep (Nitzsche et al., 2015), ferret (Hutchinson et al., 2017) and marmoset (Liu et al., 2018). Anatomic MRI atlases for the equine brain, have been published both with (Kimberlin et al., 2017) and without (Arencibia et al., 2001) gross anatomic correlation however these atlases do not provide stereotaxic templates or volumetric datasets of anatomic regions limiting the potential used in neuroimaging research.

In this study, we document the creation of a high-resolution stereotaxic population average brain atlas of the neurologically normal equine. This atlas includes a T1-weighted brain template, tissue segmentation maps (TSMs) for white matter (WM), gray matter (GM) and cerebrospinal fluid (CSF), and segmented priors of the subcortical brain structures. We document mean volume metrics for the whole brain, GM and WM and assess the effect of age and laterality on tissue volumes. The resulting atlas is correlated to gross anatomical specimens and is made freely available at as an online resource for researchers.

## Materials and Methods

### Subjects

*Ex-vivo* equine cadaver brains were recruited from the necropsy floor from animals previously euthanized for non-neurological purposes unrelated to this study. Subjects were required to have had no history of neurological disease, be between 18 months and 30 years of age and measure between 140 and 180 cm at the withers. Cadavers were prepared within 2 h of euthanasia and decapitation performed at the level of the atlanto-occipital articulation. The dura was clamped with a tie and transected to prevent cerebrospinal fluid leakage and gas tracking into the cranium. All specimens were handled and transported according to institutional biosecurity recommendations. As this study only utilized *ex-vivo* specimens harvested from animals euthanized for reasons unrelated to this study, no institutional animal care and use approval was required.

### MRI Examination

Brains were imaged *ex-vivo* and *in situ* within the cranium within 4 h of euthanasia. Imaging was performed in a GE Discovery MR750 3.0T MRI scanner with the use of a 16 channel Flex NeoCoil that was wrapped around the dorsal aspect of the cranium. A T1-weighted 3-dimensional sequence [magnetization-prepared 180 degrees radio-frequency pulses and rapid gradient-echo (MPRAGE)] was performed with the following parameters; repetition time = 7.364, echo time = 3.468, inversion time = 425, averages 1, matrix = 256 × 256, spatial resolution = 1 mm^3^. T1-weighted examinations were screened for obvious structural abnormalities by a board-certified veterinary radiologist prior to being included in the data analysis. Any subject with obvious structural abnormalities was excluded.

### Subject Demographics

Ten equine cadavers were harvested and imaged for generation of the T1-weighted template. On subjective evaluation of the scans, one subject had evidence of a pituitary mass and was excluded. The final nine subjects included two mares, and seven geldings aged between 3 and 23 years of age (mean 12.7 years old, standard deviation 8.6), two subjects were classified as adult but had no known specific age. An additional five equine cadavers were used for intersubject variability testing of the average T1 template. The signalment of each individual used in this study is documented in Table 1.

**Table 1:** Demonstrates the age, sex and breed of each subject included in the group that formed the final template (template cohort) and the group that was used for inter-subject variability testing [validation cohort; n, number of subjects, Mn, male neutered (gelding), and Fe, female entire].

Subject	Age (years)	Sex	Breed
Template Cohort (*n* = 9)
1	22	Mn	Quarter horse
2	Adult	Mn	Mustang
3	5	Mn	Standardbred
4	3	Mn	Standardbred
5	4	Mn	Quarter horse
6	Adult	Mn	No specified
7	17	Fe	Warmblood
8	15	Fe	Warmblood
9	23	Mn	Warmblood
Validation Cohort (*n* = 5)
1	Adult	Mn	Thoroughbred
2	4	Fe	Quarter horse
3	4	Fe	Thoroughbred
4	2	Mn	Thoroughbred
5	2	Mn	Thoroughbred

### Data Processing

**Figure 1:**
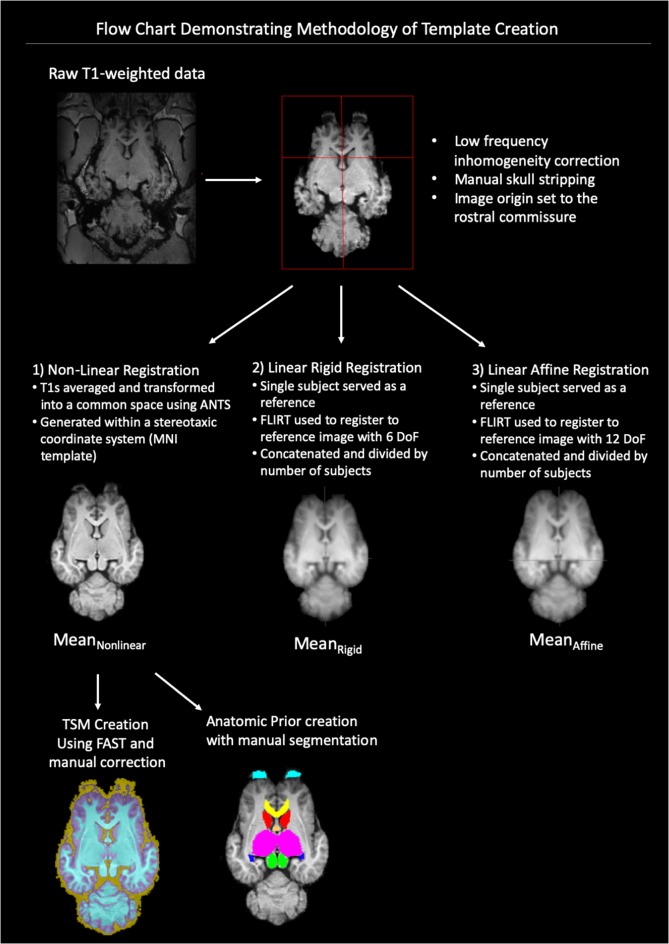
Demonstrates a flow chart of the methodology we used for data processing and template creation. Raw T1-weighted data underwent low frequency inhomogeneity correction before manual removal of non-brain tissue and standardization of image origin to the rostral commissure. The data then underwent three types of registration non-linear, linear rigid and linear affine. The final template was created from the Mean _Non-linear_ registration template and tissue segmentation maps (TSMs) and anatomic priors created (ANTs, advanced normalization tools, FAST, FMRIB’s automated segmentation tool, MNI, Montreal Neurological Institute).

A flow-chart depicting the methodology of the pre-processing and template creation is provided in Figure 1.

### Pre-processing

A subset of equines was used to create the T1-weighted template. The MRI data were corrected for low frequency intensity inhomogeneity (Tustison et al., 2010). A combined approach of automated (Smith, 2002) and manual removal of non-brain tissues was applied prior to the images being affine registered (Smith et al., 2004) and spatially normalized (Friston et al., 1995). The origin of the images was set to the rostral commissure, then the data were reoriented to a standard FMRI Software Library (FSL) orientation for consistency (Jenkinson et al., 2012). The data were then trimmed (removal of non-brain space from the image) and set to standard dimension for all images.

### Template Creation

All the individual subjects’ T1 data were then transformed into a common space population template (Mean_Non-linear_) using the build template script from Advanced Normalization Tools (ANTs) which uses symmetric normalization (SyN), diffeomorphism and affine transformation to produce a group mean size and shape (Avants et al., 2011). This template was generated with a stereotaxic coordinate system according to Montreal Neurological Institute (MNI) template specifications and in line with other animal templates (Nitzsche et al., 2015). The origin of the Cartesian system (x, y, z; 0, 0, 0) was centered on the mid-line over the dorsal aspect of the rostral commissure. The zero x-axis value sagittal plane extended through the center of the brain in line with the falx cerebri, the zero y-axis value transverse plane was parallel to the anterior commissure and transected the brain symmetrically and the zero z-axis value dorsal plane ran from the dorsal rostral commissure to the mesencephalic aqueduct, ventral to the caudal commissure. Sagittal plane x-axis values increased left to right, transverse plane y-axis values increased caudal to rostral and dorsal plane z-axis values increased ventral to dorsal. All co-ordinates are provided in millimeters.

### Template Quality Assessment

For quality assessment purposes, templates using both linear rigid and affine registration were created from the subject data for comparison to the Mean_Non-linear_ template. After the pre-processing procedure described above, a single subject was chosen at random to serve as a reference for the linear templates. For the creation of a rigid registration average FMRIB’s Linear Registration Tool (FLIRT) was used to register each subject to the reference image with 6 degrees of freedom (DoF; Jenkinson and Smith, 2001; Jenkinson et al., 2012). These registered images were concatenated and then divided by the number of subjects to create a linear rigid mean template (Mean_Rigid_). Similarly, FLIRT was used to create a linear affine mean template (Mean_Affine_) by registering each subject to the reference image with 12 DoF then concatenating and dividing in the same fashion (Jenkinson and Smith, 2001; Jenkinson et al., 2012). The standard deviation for each voxel was calculated from each of the comparison templates Mean_Rigid_ and Mean_Affine,_ and the non-linear template—Mean_Non-linear_. The quality of each template and subject volumes were assessed subjectively and quantitatively. Signal-to-noise (SNR) and contrast-to-noise (CNR) ratios were calculated using the widely accepted equations (Allen et al., 2003). The data were tested for normality. The SNR data were found to have a parametric distribution and so a one-way analysis of variance (ANOVA) test was selected to examine the differences in GM SNR and WM SNR between registration templates (Mean_Rigid,_ Mean_Affine_ and Mean_Non-linear_). The CNR data across each template were found to be non-parametric in nature, with significant kurtosis, and therefore the a Kruskal–Wallis test was used to examine differences in CNR across registration templates (Mean_Rigid,_ Mean_Affine_ and Mean_Non-linear_).

### Tissue Segmentation Maps (TSMs)

TSMs were created for each subject and template using FMRIB’s Automated Segmentation Tool (FAST) which segments brain matter into cerebral spinal fluid, GM, and WM while correcting for spatial intensity variations (Zhang et al., 2001). FAST was used to create partial volume maps, TSMs of each tissue type, binary segmentation masks and bias field maps. Binary segmentations were assessed anatomical coherence with the T1 weighted scan. It was noted that FAST had overestimated the cerebral spinal fluid and WM masks and these masks were manually corrected to more accurately correspond to the T1 neuroanatomy. FAST was then re-run with the corrected tissue segmentation masks for the creation of corrected partial volume masks and TSMs. The corrected partial volume masks were used to calculate the tissue volume to account for partial volume effects and increase sensitivity.

### Anatomic Prior Creation

From the population template anatomically significant regions were manually delineated from anatomic and imaging references (Arencibia et al., 2001; Pascalau et al., 2016; Kimberlin et al., 2017) using mask creating software FSLeyes (version 027.0 FMRIB Center, Oxford, UK). Segmented regions were created only for those boundaries which were discernable on the T1-weighted imaging sequences and included; olfactory bulbs, rostral commissure, caudate nuclei, globus pallidus, thalamus, hypothalamus, optic chiasm, pineal gland, corpus callosum, fornix, hippocampi, amygdala, mesencephalon, pons, medulla oblongata and cerebellum. Segmentations were performed by a board-certified diplomat in veterinary radiology (PJ), who has expertise in neuroimaging and reviewed by a veterinary neuroanatomist (MF).

### Correlation of Volume With Age and Assessment of Hemispheric Laterality

The GM, WM and whole brain tissue volumes (mm^3^) were calculated for each subject and correlated to age using a Pearson correlation in the statistical software SPSS [correlation being significant at the 0.01 level (2-tailed)]. In order to investigate potential asymmetries in the equine brain, individual subjects and the Mean_Non-linear_ population average template were visually inspected and the midline slice in the x-axis was used to delineate the volume into left and right hemisphere using FSLUTILS (Allen et al., 2003). The partial volume masks of GM and WM from FAST were divided by the midline and the tissue volumes (mm^3^) for GM and WM were calculated using FSLUTILS (Allen et al., 2003). The data were tested for normality and an independent *t*-test performed to determine significant differences between hemispheres using the statistical software SPSS (significance considered present with a *p*-value < 0.05). Laterality indices were calculated according to the following formula from Boltze et al. (2018):

$$I^{lat}=V_{contralateral}^{tissue}/V_{ipsilateral}^{tissue}$$

The lateralization index for GM and WM was calculated using left as contralateral and right as ipsilateral.

### Brain Tissue Processing

After imaging, brains were extracted from the cranium and underwent fixation *via* formalin submersion. Brains were immersed in approximately two liters of 10% neutral-buffered formalin for at least 7 days. The brains were stored in formalin until the time for processing (between 4 and 8 months). For processing and image creation, gross whole brains were photographed in lateral and dorsal. Brains were then sliced either in the mid-sagittal plane or in transverse plane at the level of the caudate nuclei, rostral commissure, hypothalamus, hippocampi and pons and all slices photographed. The most representative images were used and correlated to the associated surface anatomy or template level and priors.

### Inter-subject Variability Testing

In order to assess, how well our atlas assists with registration and normalization of brain data, five additional equine data sets were recruited. These independent testing samples were aligned to the bi-commissural line of the equine template using rigid-body 6 DoF and the differences in brain shape and structure visually assessed. The testing samples were then registered to the equine template using both 12 DoF linear registration using FLIRT and non-linear warping using ANTs. The different registrations were evaluated by visually comparing the structural differences between subjects. In order to more quantifiably assess variation between subjects after each 6 DoF rigid-body alignment, 12 DoF linear registration and non-linear warping voxel-wise standard deviation across subjects was calculated and plotted on brain maps. Pearson’s correlation coefficients were then computed for every combination of the T1 volumes after 6 DoF rigid-body alignment, 12 DoF linear registration and non-linear warping and differences tested for significance with a one-tailed *t*-test.

## Results

### Template Quality Assessment

**Figure 2:**
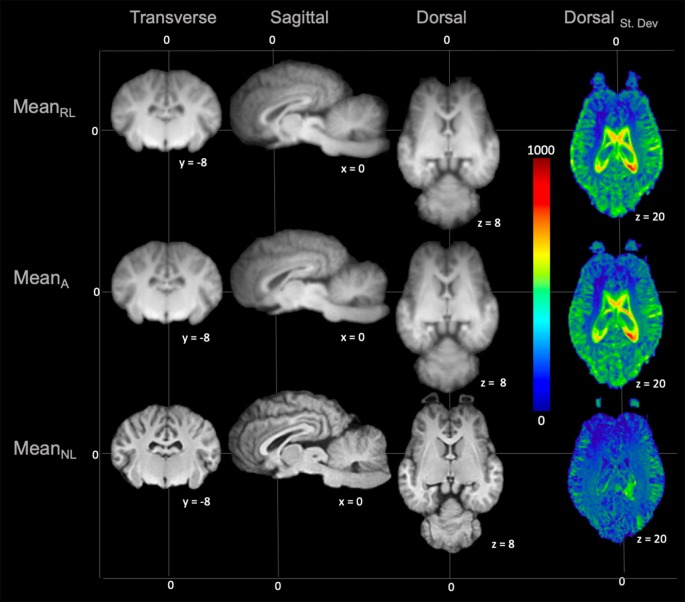
Demonstrates the rigid linear (Mean_RL_), affine (Mean_A_) and non-linear (Mean_NL_) templates in mid transverse, mid sagittal and mid dorsal planes. The non-linear template (Mean_NL_) has clearer boundaries between structures and exhibits better GM and WM definition, when compared to the rigid linear (Mean_RL_) and affine (Mean_A_) templates. The corresponding standard deviation (blue = 0 and red = 1,000) for each template is demonstrated in mid dorsal plane on the right column. This shows that there is a reduced standard deviation in the non-linear (Mean_NL_) template than the other templates.

**Figure 3:**
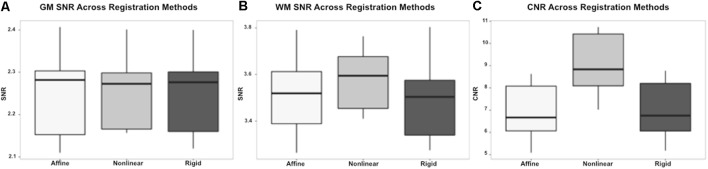
Box and whisker plots demonstrating the signal to noise ratio (SNR) and contrast to noise ratio (CNR) for the different registration methods. **(A)** Demonstrates the SNR for gray matter (GM) which is similar between methods, with no statistically significant difference. **(B)** Demonstrates the SNR for white matter (WM) which is higher in the non-linear registration method, though no statistical difference was identified. **(C)** Demonstrates the CNR of the brain for each registration methods and shows that the non-linear method has the highest CNR. This difference was statistically significant.

On subjective evaluation of the created templates, no significant artifact was found to impact image quality. The non-linear template had clearer boundaries between structures, exhibited good GM and WM definition, and had consistently higher SNR and CNR (Figure 2). Statistically, there were no significant differences in GM SNR or WM SNR between templates (*F*_(2,24)_ = 0.08, *p* = 0.92). There was a significant difference found in CNR (Chi square = 8.95, *p* < 0.05, *df* = 2) with the Mean_Non-linear_ template having the highest mean CNR (9.03), and the Mean_Rigid_ (7.09) and Mean_Affine_ (7.01) each having lower mean CNR (Figures 3A–C).

### TSM’s, Brain Surface Architecture and Anatomic Priors

**Figure 4:**
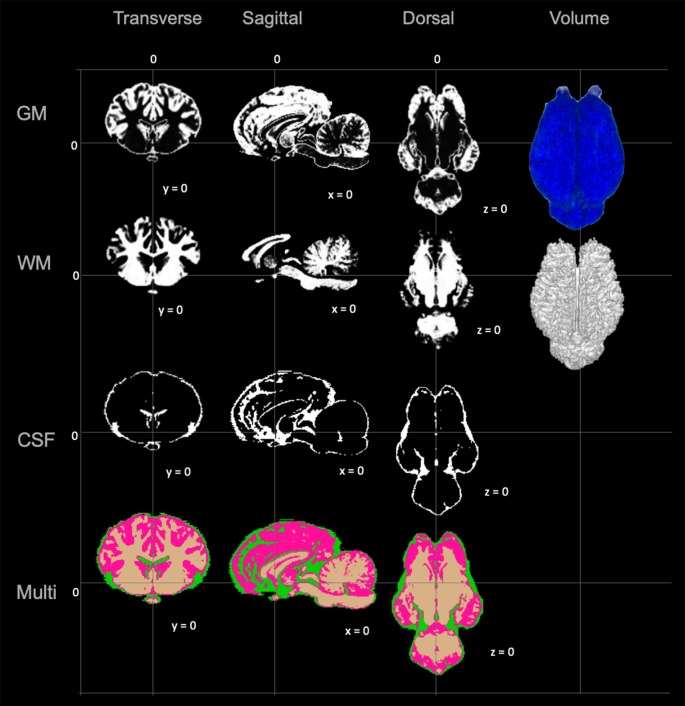
Visual demonstration of the TSMs. The gray matter (GM) map, white matter (WM) map, cerebrospinal fluid (CSF) map and a combination of all maps (Multi) are demonstrated in mid transverse, mid sagittal and mid dorsal planes. Volume rendered images for the GM and WM maps are also included in the right column.

**Figure 5:**
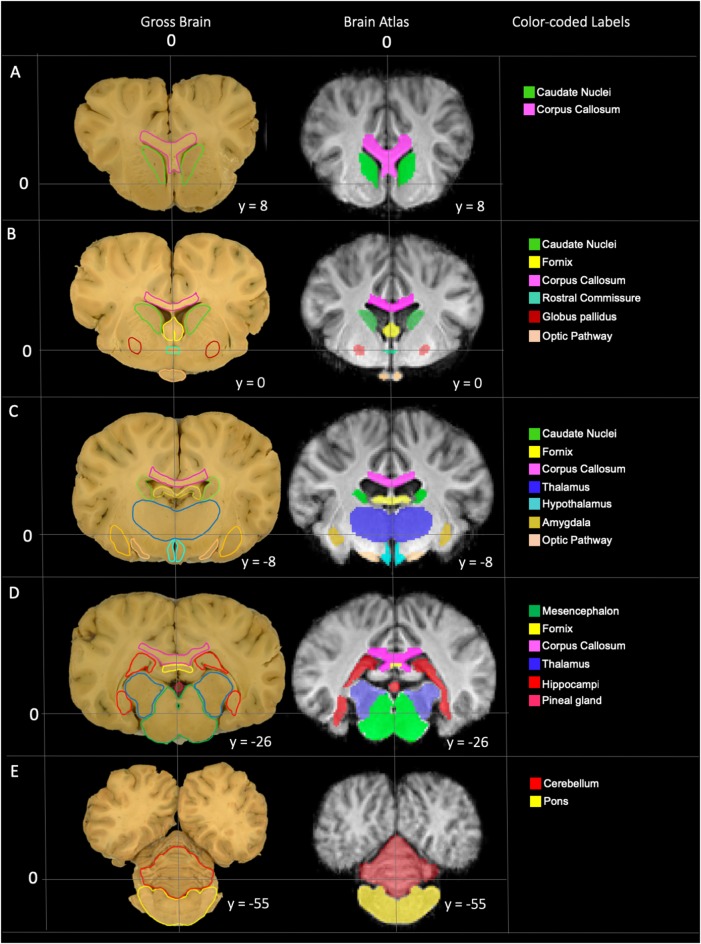
Labeled images demonstrating the manually segmented anatomic priors with correlation to an anatomic specimen. **(A)** Transverse plane slice at the level of the caudate nuclei (green) demonstrating the rostral aspect of the corpus callosum (pink). **(B)** Transverse plane slice at the level of the globus pallidus (maroon) including the caudate nuclei (green), corpus callosum (pink), rostral fornix (yellow), rostral thalamus (dark blue), rostral commissure (turquoise) and optic pathway (peach). **(C)** Transverse plane slice at the level of the inter-thalamic adhesion including the mid corpus callosum (pink), caudal caudate nuclei (green), mid fornix (yellow), mid thalamus (dark blue), hypothalamus (light blue), amygdala (dark yellow) and caudal optic pathway (peach). **(D)** Transverse plane slice at the level of the hippocampi (red) including the caudal corpus callosum (pink), caudal fornix (yellow), caudal thalamus (dark blue), pineal gland (dark pink), and mesencephalon (dark green). **(E)** Transverse plane slice at the level of the pons (yellow) including the cerebellum (red).

**Figure 6:**
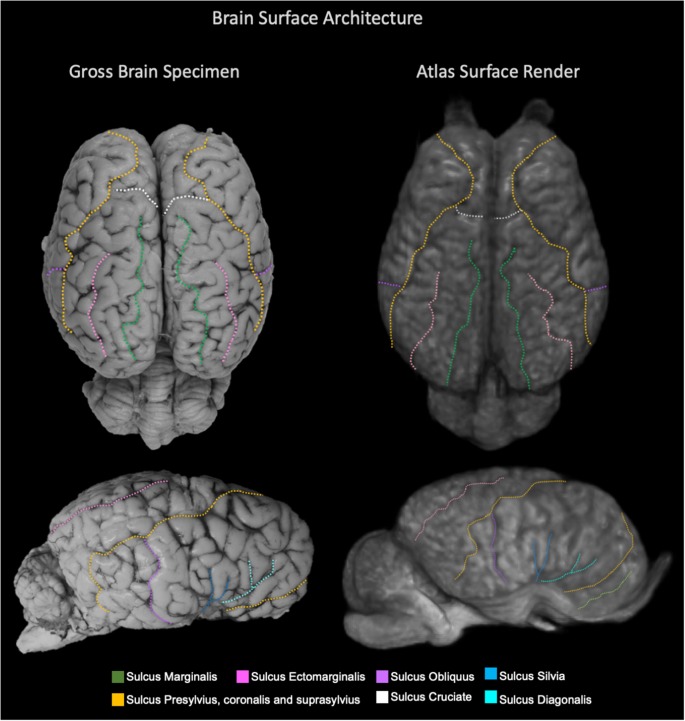
Labeled images demonstrating the surface architecture of the atlas (right) in comparison with an anatomic specimen (left). Images of the dorsal (top) and lateral (bottom) brain surfaces are provided and the sulci are labeled. Sulci included; marginalis (green), ectomarginalis (pink), obliquus (purple), silvia (blue), presylvius, coronalis and suprasylvius (yellow), cruciate (white) and diagonalis (turquoise).

The TSMs created from the non-linear template are demonstrated in Figure 4. The manually segmented anatomic priors of the olfactory bulbs, rostral commissure, caudate nuclei, globus pallidus, thalamus, hypothalamus, optic chiasm, pineal gland, corpus callosum, fornix, hippocampi, amygdala, mesencephalon, pons, medulla oblongata and cerebellum are compared to corresponding transverse gross brain slices (Figure 5). The surface render of the brain atlas is compared to a gross brain specimen and corresponding sulci outlined according to the recent anatomic literature (Figure 6; Lang et al., 2018).

### Tissue Volume in Relation to Age and Laterality

**Figure 7:**
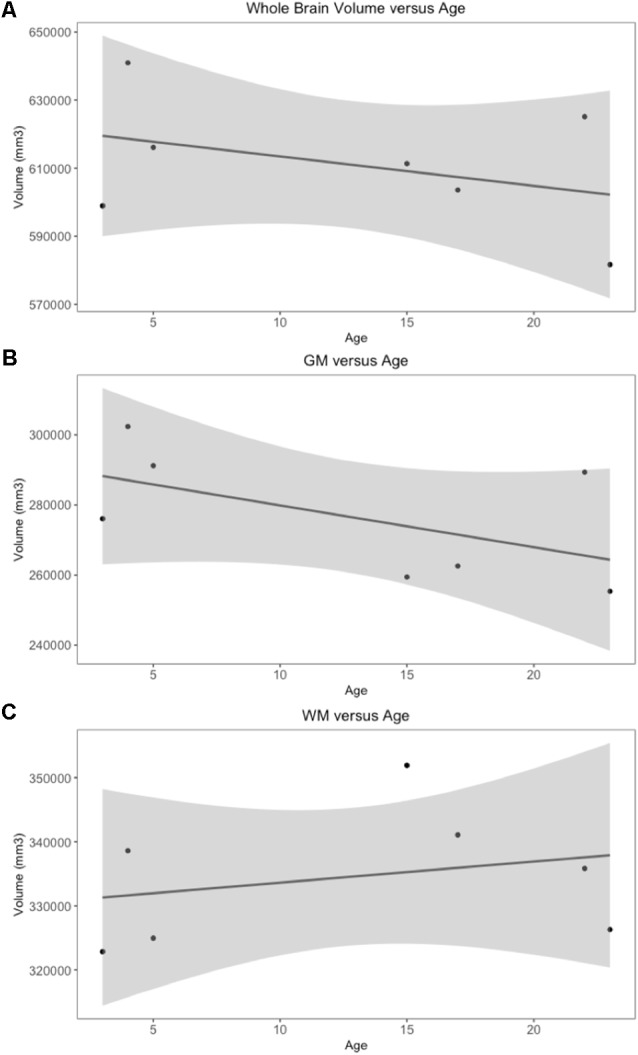
Scatter plots of individual subject whole brain, gray matter (GM) and white matter (WM) volumes plotted against age. Only subjects with a definitive age (subject 1, 3, 4, 5, 7, 8 and 9) were included in this analysis). (**A**) Whole brain volume vs. age demonstrates a downward trend in volume as age increases. **(B)** GM vs. age demonstrates a downward trend in volume as age increases. **(C)** WM vs. age demonstrates an upward trend in volume as age increases.

**Figure 8:**
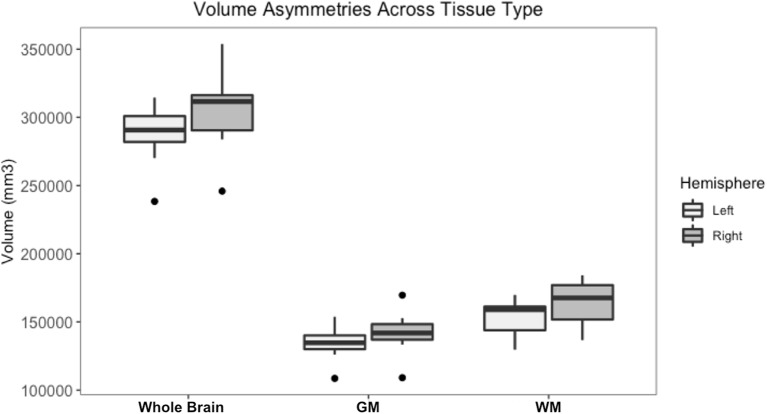
Box and whisker plots of right and left hemisphere volumes of the whole brain gray matter (GM) and white matter (WM). All subjects had higher right-sided volumes for all tissue types however an independent *t*-test found no statistically significant difference between right and left sides for whole brain (*p*-value = 0.75), GM (*p*-value = 0.212) or WM (*p*-value = 0.104, significance considered present with a *p*-value < 0.05).

Whole brain and GM volume trended down with increasing age, whereas WM volume trended up however a statistically significant correlation between age and tissue volume was not identified [Figure 7; correlation being significant at the 0.01 level (2-tailed)]. In all subjects, GM and WM volumes were higher on the right side. The data had a normal distribution when evaluated with a Shapiro–Wilk test. The independent *t*-test found no statistically significant difference between right and left sides for whole brain (*p*-value = 0.75), GM (*p*-value = 0.212) or WM (*p*-value = 0.104, significance considered present with a *p*-value < 0.05; Figure 8). The laterality index for the WM was 0.96 and for the GM was 0.98 indicating that, although a high level of matching was present between hemispheres, in both tissues the right side was larger.

### Inter-subject Variability Testing

**Figure 9:**
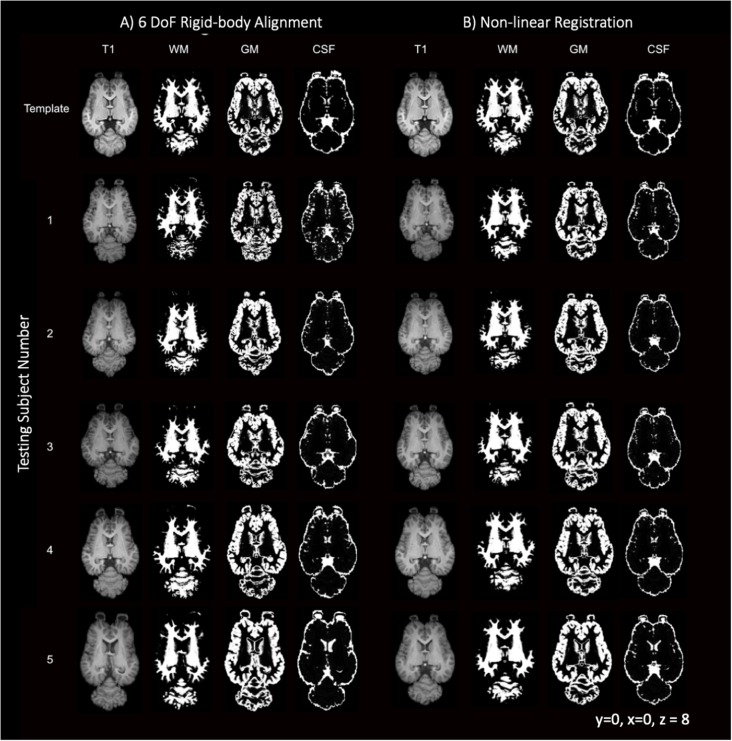
T1-weighted, GM, WM and CSF slices of the template and each individual testing subject. **(A)** Demonstrates the template (top row) and each of the five testing individuals after 6 degrees of freedom (DoF) rigid body alignment to the template. **(B)** Demonstrates the template (top row) and each of the testing individual subjects after non-linear registration to the template.

Like most mammals, the equine brain shows some variability in size, shape and cortical morphometry. The variability of the subjects is visually apparent when comparing individuals in Figure 9 with subject 5 exhibiting mildly enlarged lateral ventricles and subjects 3 and 5 having variation in lateral and rostrocaudal lengths. The brain size and cortical variation was reduced following non-linear registration and are visually apparent in Figure 9 with the normalization of ventricles and cortex across subjects.

**Figure 10:**
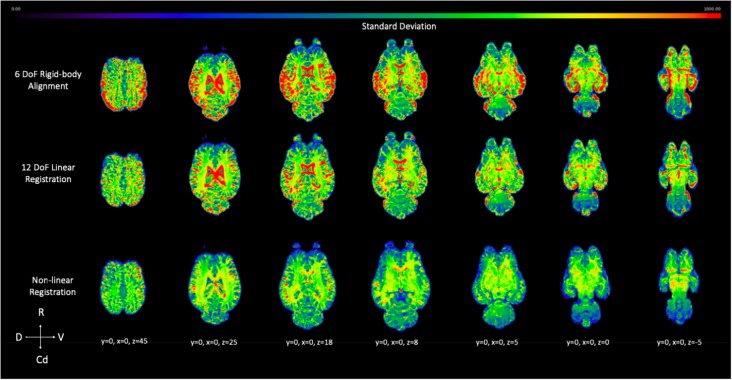
Demonstrates the effect of 12 DoF linear registration and non-linear registration on the inter-subject spatial variability. The voxel intensity within the spatial maps represents the degree of standard deviation for the five testing subjects after 6 DoF rigid body alignment (top row), 12 DoF linear registration (middle row) and non-linear registration (bottom row) to the template (R, rostral; Cd, caudal; D, dorsal; V, ventral).

The voxel-wise standard deviation across subjects was calculated for the 6 DoF rigid-body alignment, 12 DoF linear registration and non-linear registration of the testing sample (Figure 10). While there was a reduction in variation in the linear registration, the least amount of variation, and lower standard deviation (blue), across subjects was achieved with non-linear warping as demonstrated visually in Figure 10.

Pearson’s correlation coefficient testing identified that linear (*M* = 0.95, SD = 0.01) and non-linear (*M* = 0.98, SD = 0.002) registration both significantly improved the matching of brain volumes to the equine T1 template over the alignment procedure (*M* = 0.92, SD = 0.02; linear > aligned: *t*_(18)_ = −5.59, *p* < 0.05 ; non-linear > aligned: *t*_(18)_ = −11.40, *p* < 0.05). In addition non-linearly registered images to the T1 template were significantly more correlated than those linearly registered (non-linear > linear: *t*_(18)_ = −16.34, *p* < 0.05). These results indicate that non-linear registration most accurately normalized the testing equine sample into the equine T1 template space.

## Discussion

The objective of this study was to create an anatomically correlated, standard stereotaxic brain atlas for the horse for use in neuroscientific and equine clinical research. The template was created from nine equine cadaver subjects with their brains imaged *in situ* within the cranium. The brain tissue volumes of the individual subjects altered according to age and all horses exhibited insignificantly higher volumes on the right side. Quality assessment identified that the non-linear population average template, created from ANTs, had better signal and contrast to noise ratios than corresponding rigid linear and affine templates. From this template TSMs and manually segmented volume priors of several subcortical regions were generated.

This atlas joins a battery of stereotaxic brain atlases available for multiple other species, including the cat (Stolzberg et al., 2017), dog (Datta et al., 2012), sheep (Nitzsche et al., 2015), ferret (Hutchinson et al., 2017) and marmoset (Liu et al., 2018). These atlases have been created from variably sized animal cohorts using similar 3-dimensional T1-weighted MR images. In our study, the non-linear template created with the use of ANTs was superior to rigid linear and affine templates. This correlates with that described in other atlases and ANTs is a well-accepted and commonly used method for template generation (Datta et al., 2012; Nitzsche et al., 2015).

The atlas was created from nine cadaveric subjects whose un-fixed brains were imaged *in situ* within the cranium within 4 h of euthanasia. This method has been described in post-mortem fetal MRI studies and minimizes post mortem tissue autolysis and distortion of the brain which would have occurred if it had been removed prior to imaging (Scola et al., 2018). It is important to note, however, that despite these precautions, structural brain changes can occur secondary to a lack of blood pressure post-mortem and therefore cannot be considered the identical to *in vivo* clinical imaging data.

Laterality within the brain reflects hemispheric brain activity and processing. In horses motor, sensory laterality is present at an individual level, and a strong right forebrain dominance has been identified when horses process stressful, agonistic and social interactions (Larose et al., 2006; Austin and Rogers, 2007; Farmer et al., 2010, 2018). Our results support a right brain dominance with all our subjects exhibiting higher whole brain, GM and WM volumes on the right side. Prior to euthanasia, our subjects did not undergo motor or sensory laterality evaluations and so correlation between behavior and imaging was not possible.

The limitations of this atlas include the low subject number and the uneven sex distribution. As an average population template the larger the number of subjects included, the more representative the atlas is to the general population. Our atlas contained nine subjects, which is similar to that of other brain atlases (Datta et al., 2012; Stolzberg et al., 2017); however, this could result in some bias from the true average. Additionally, our cohort had more geldings than mares and included no stallions. This limited our ability to test the effect of sex and neutered status on brain tissue volume. Sexual dimorphism has been observed in human GM and WM (Allen et al., 2003); however, significant differences were not identified in an evaluation of ovine brains (Nitzsche et al., 2015) and so the true impact of this limitation on the resultant brain atlas remains unclear. The anatomic images were created from a representative neurologically normal equine subject, however the signalment of the subject is unknown.

## Conclusion

Here, we present an anatomically correlated, standard T1-weighted stereotaxic brain atlas for the horse, which includes TSMs for CSF, GM and WM, and manually segmented anatomic priors for the olfactory bulbs, rostral commissure, caudate nuclei, globus pallidus, thalamus, hypothalamus, optic chiasm, pineal gland, corpus callosum, fornix, hippocampi, amygdala, mesencephalon, pons, medulla oblongata and cerebellum. This atlas is made freely available in NIFTI-1 format at: https://doi.org/10.7298/cyrs-7b51.2.

## Data Availability Statement

All data sets generated for this study are included in the manuscript and made freely available in NIFTI-1 format at: https://doi.org/10.7298/cyrs-7b51.2.

## Ethics Statement

This study involved the use of animal tissue after euthanasia and was exempt from ethics approval.

## Author Contributions

The following parts of the study were performed by: PJ and VJ: study design; PJ, VJ and W-ML: data acquisition; EB and PJ: data processing; MF, TS and PJ: neuroanatomic correlation; PJ, EB, VJ, MF, TS and W-ML: manuscript preparation and evaluation.

## Conflict of Interest

The authors declare that the research was conducted in the absence of any commercial or financial relationships that could be construed as a potential conflict of interest.
